# Oral reconstruction with hybrid implant‐supported fixed prostheses in cases of mandibular defect using two methods along with 3 years of follow‐up: A case report

**DOI:** 10.1002/ccr3.4854

**Published:** 2021-09-24

**Authors:** Somayeh Niakan, Negin Yaghoobi

**Affiliations:** ^1^ Dentistry Research Institute Department of Prosthodontics Dental Research Center Tehran University of Medical Science Tehran Iran

**Keywords:** ameloblastoma, dental implant, dental prostheses, implant‐supported prosthesis

## Abstract

Ameloblastoma is an invasive odontogenic tumor, and for reconstruction, iliac graft and dental implants play an important role. This article evaluates the application of hybrid prostheses, clinical steps, and complications related to this treatment.

## Graphical abstract

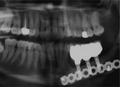

## 1 INTRODUCTION

Hybrid Prosthesis is considered as a rational treatment option that offer both advantages of cement‐retained and screw‐retained implants. This study presents a series of two cases of ameloblastoma which received iliac graft after resection treated by Hybrid Prosthesis and were observed for 3 years.

Ameloblastoma accounts for the second most frequent odontogenic tumor after odontoma, and it is considered as a tumor with odontogenic epithelial origin.^1^ This tumor is more common in the angle and mandible body, and infrequent cases have been observed in maxilla which are much worse than mandible due to the greater risk of penetration in the trabecular bone in this site.^2^, ^3^

The resection of this tumor may cause some impairments such as difficulty in speech, swallowing, saliva retention, deficiency in other functions, obvious facial deformity, and emotional and psychological problems. Furthermore, the destruction of alveolar bone and deterioration of teeth can lead to inadequate mastication ability. The aim of performing reconstruction in patients with these defects by employing autogenous bone grafts or revascularized free flaps is to increase the probability of bone continuity and facial contour so that one can provide the location for inserting oral implants and overcome problems such as unsatisfactory functions and esthetic issues.^2^, ^4^

In order to reconstruct the mandibular defects, autogenous bone grafts which are taken from the ileum or calvarium are reliable and useful. They provide sufficient support for the placement of implants and implant‐supported prosthetic restorations and recreate facial contour as well. Chiapasco et al. in 2008 evaluated the survival rate of implants in resected area of mandible after reconstructing with autogenous graft. They concluded that the calvarium or anterior iliac crest which are used for the rehabilitation of the affected area meet a high survival rate (96.7%) after a long follow‐up period (mean value of 94 months, ranging from 36 to 132 months).^4^ In comparison with the calvarium, the iliac graft contains corticocancellous structure and multipotential cells capable of providing adequate bone height and bulk for the reconstruction of mandibular defects in order to compensate discontinuity of the mandible and replace facial contour.^4^, ^5^ Based on the evidence of invasion in patients with mandibular ameloblastoma who received iliac bone graft, rare cases of recurrence have been reported many years after the initial operation.^6^

Implant‐supported prostheses can be divided into removable and fixed restorations (cement‐retained, screw‐retained, and hybrid restorations). “Hybrid restorations” describes a screw‐retained framework with cement‐retained crowns used as a treatment option that offer both advantages of cement‐retained and screw‐retained implant prostheses. Therefore, these types of restorations are considered as a prosthesis of choice for the reconstruction of moderately to severely resorbed alveolar ridges.^7^, ^8^ Fixed hybrid prostheses splint implants together, provide adequate strength using well‐designed framework, and fulfill esthetic demands. Furthermore, retrievability, passive casting, proper hygiene, simple maintenance and reduced fatigue, fracture or distortion of components are some of the characteristics of the hybrid restorations.^9^, ^10^, ^11^, ^12^

The purpose of this study was to present two cases referred to our department who had received iliac graft after an ameloblastoma resection and partial mandibulectomy. Due to the increased interocclusal space and patients demands, it was decided to use fixed hybrid implant‐supported prostheses in order to benefit from both advantages of cement‐retained and screw‐retained restorations.

## 2 CASE HISTORY

### 2.1 Case 1

A 14‐year‐old boy with deficiency in left side of mandible was referred to our department. The patient's chief complaint was mastication problem, difficulty in speech and swallow.

**FIGURE 1:**
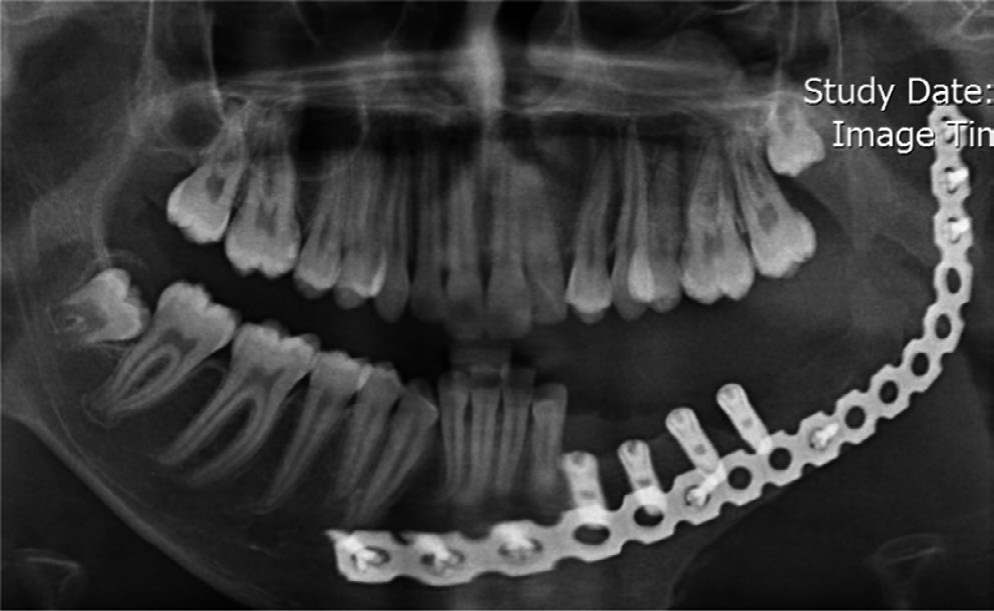
Noticeable bone resorbtion and failed implant at the site of #18

Four implants (Implantium, Dentium) were inserted into the planned sites. However, during the healing periods, one of the implants failed, and after 6 months, three implants (3.8 × 12), which were placed at the #19, #20, and #21 sites, were prepared for the prosthetic procedures (Figure 1).

The fixture‐level impression was made with polyvinyl silicone impression material (Panasil, Kettenbach GmbH & Co., KG) using a stock tray and splinted open‐tray impression copings. Also, an irreversible hydrocolloid impression (Chromogel) was made of the upper jaw. A vacuum‐mixed Type IV dental stone (Hero Stone Vigodent Inc) was also used to fabricate the definitive cast.

In order to register the centric position, lower record base, occlusion rim, and interocclusal registration material (Futar^®^ D, Kettenbach GmbH& Co., KG) were used. Then, the casts were mounted in a semi‐adjustable articulator (Dentatus) using a facebow transfer record.

**FIGURE 2:**
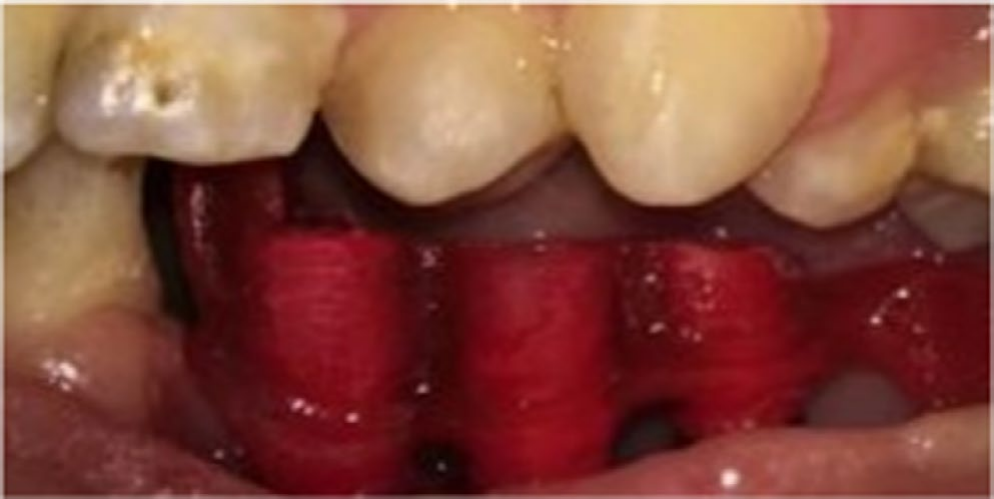
Acrylic resin pattern try in

**FIGURE 3:**
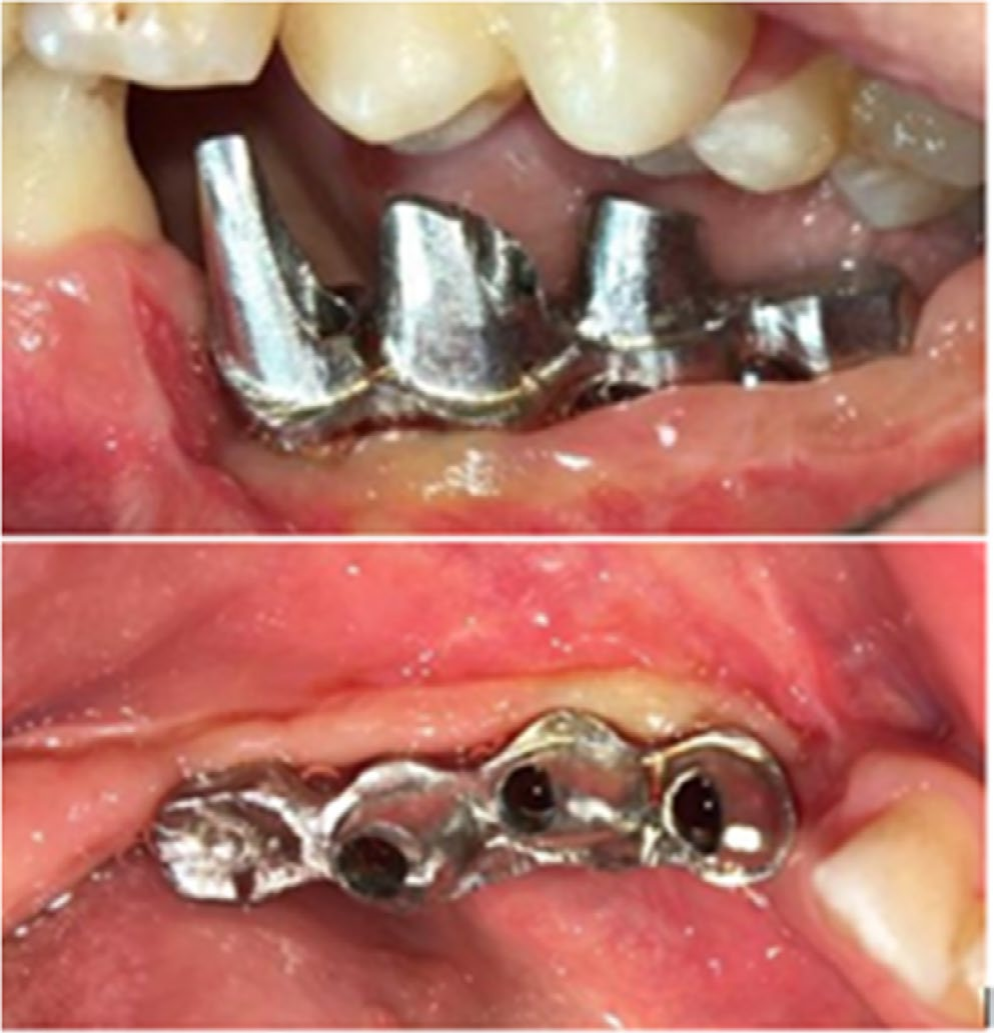
Evaluation of metal framework

After connecting the customized abutments (Metal‐Casting Abutment, SuperLine II, Dentium Co. Ltd) to the implant analogs on the master cast, the acrylic resin (Pattern Resin, GC) pattern of infrastructure (mesostructure) was made to evaluate the accuracy of impression procedure prior to casting and was tried intraorally (Figure 2). Subsequently, the mandibular metal framework casted by base metal alloys (Cr‐Co) was tried in the mouth, and adaptation was assessed by means of radiography and one‐screw test (Figure 3). Then, the pink porcelain was added to the gingival part of the framework to resemble the soft tissue in gingival areas. The copings of the crowns were directly waxed up on the framework. After cut back, metal copings were veneered by application of opaque ceramic and feldspathic ceramic. Metal‐ceramic crowns were then evaluated, and canine rise occlusion was adjusted as well. The posterior cantilever of second molar was planned by considering the occlusal relationship.

**FIGURE 4:**
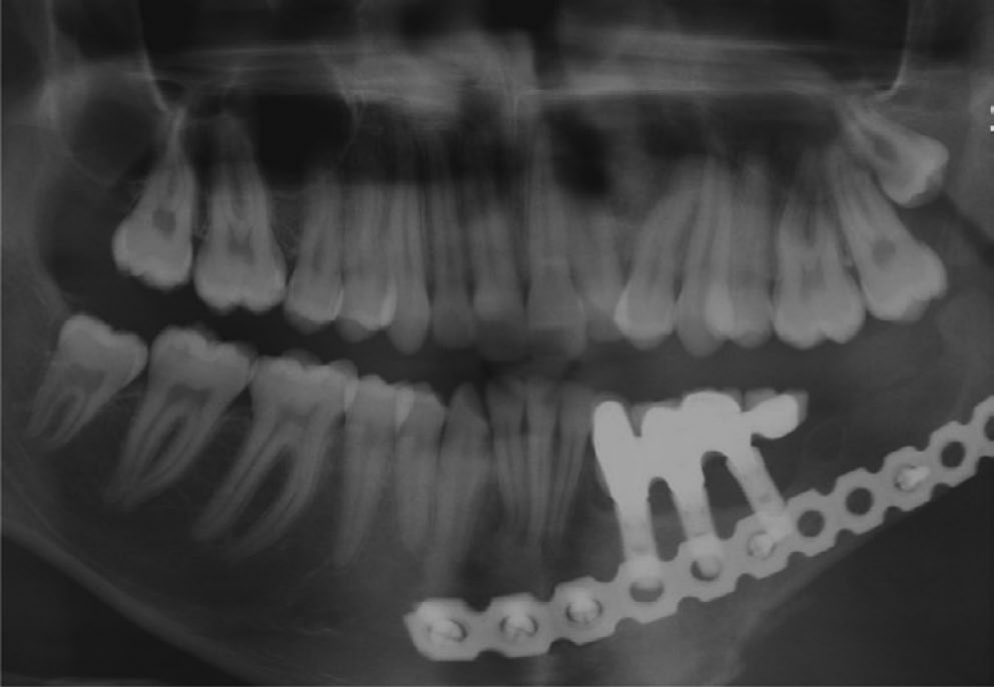
Radiograph after 3 years follow‐up

Finally, the metal mesostructure was fastened with a 30‐N cm torque, Teflon tape (SITCO) was placed at the orifice of screw holes, and crowns were cemented with temporary cement (Temp‐Bond, Kerr) on the metal framework. The baseline radiograph was taken at the end of the procedure. Moreover, the oral hygiene instructions using dental water jet and dental super floss were explained to the patient. Three years later, the patient was followed up and no functional or hygienic problems were observed, but moderate bone loss was seen around #19, #20 (Figure 4).

### 2.2 Case 2

A 22‐year‐old woman with chief complaint related to esthetics and masticatory difficulty was referred. The clinical evaluation indicated insufficient mucosal tissue and residual ridge resorption in the left side of mandible.

Three tissue level implants (4.1 × 10 mm), (Institute Straumann AG) were inserted into the #18, #19, and #21 sites.

After 4 months of healing, the fixture‐level impression was made and centric relation was recorded considering the aforementioned points in the previous case.

**FIGURE 5:**
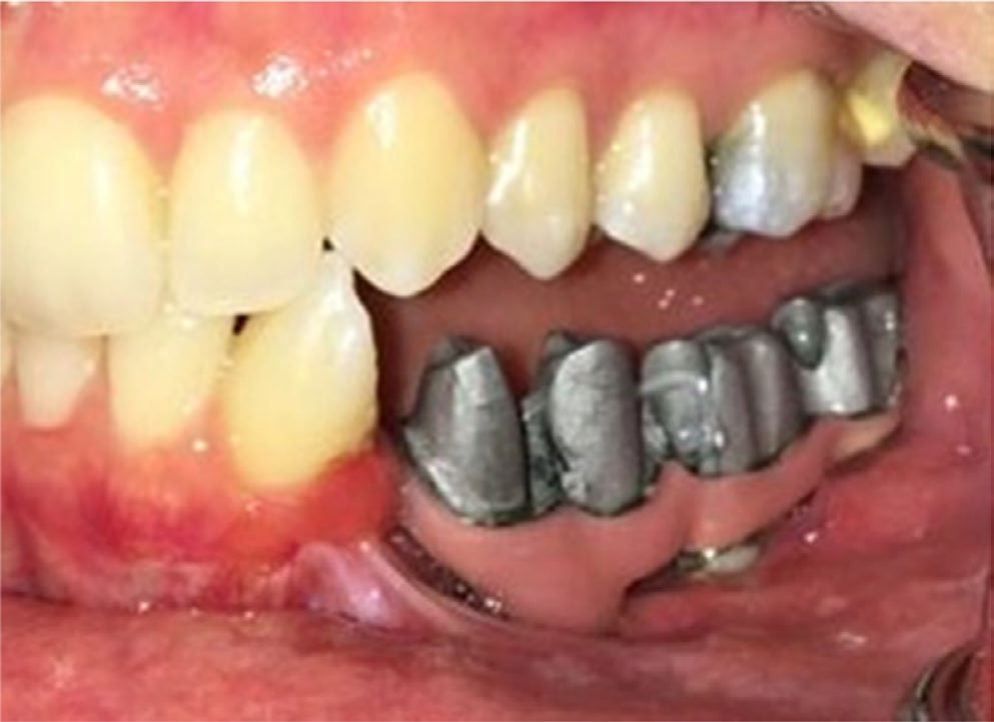
Adding pink porcelain to framework

For the definitive restoration, definitive cast was scanned (D700 scanner; 3 Shape) in the laboratory and printed abutment and mesostructured (DETAX GmbH & Co. KG) were tried in. Then, three custom abutments were virtually designed and milled with titanium blocks. The framework was designed with three screw‐access channels in order to guarantee retrievability for the maintenance and milled with Co‐Cr blank (Glorious). All the abutments were connected, and the passive fit of the framework was confirmed intraorally. Then, the pink porcelain was veneered on the gingival part of the framework and gingival level, and its contour was assessed as well (Figure 5). After veneering, the framework was scanned and four anatomic contour crowns were designed on the scanned abutment.

**FIGURE 6:**
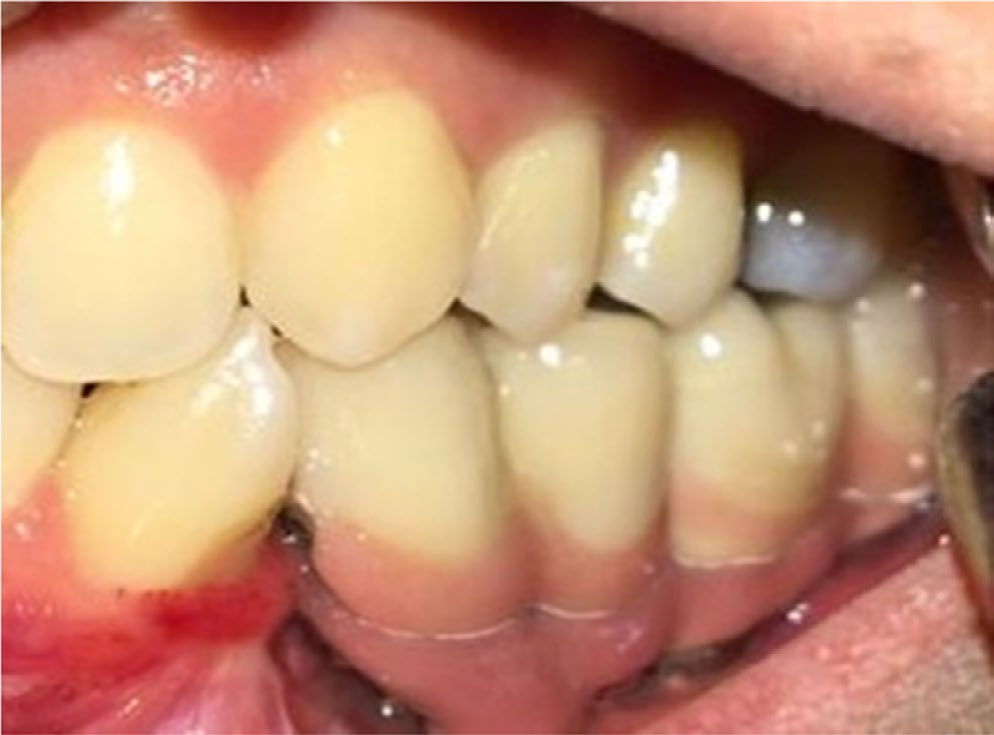
Crowns were cemented on framework

**FIGURE 7:**
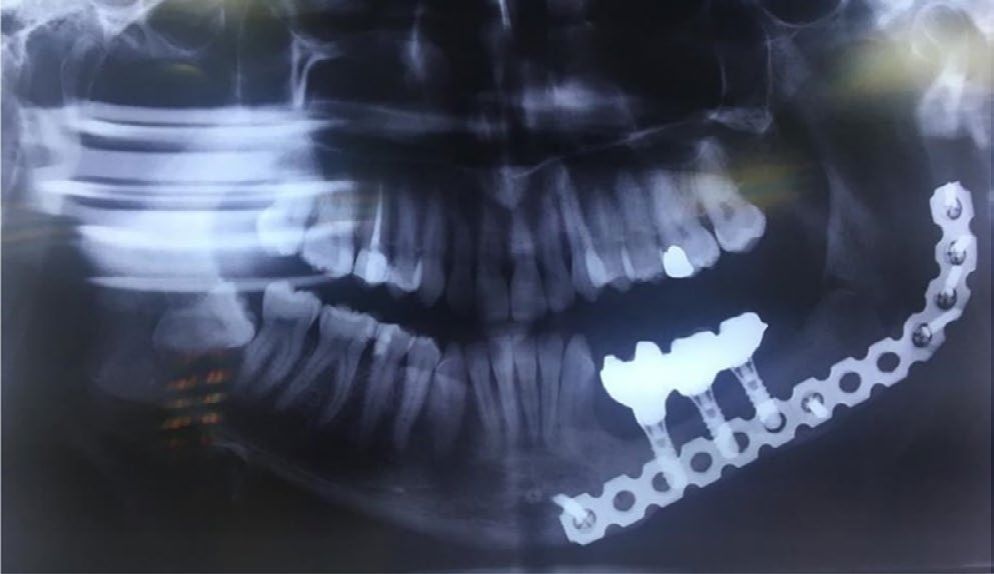
Delivery session radiograph

Finally, all abutments were connected to the fixtures with a 30‐N cm tightening torque, and the anatomic contour crowns were cemented on the metal abutment teeth (Figure 6). The hygienic points were discussed and explained to the patient and baseline radiograph was taken as well (Figure 7).

During a 3‐year follow‐up period, the screw loosening was observed in one of the abutments. One of the challenges in current case was that holes for accessing to abutments were not provided in the anatomic crowns.

**FIGURE 8:**
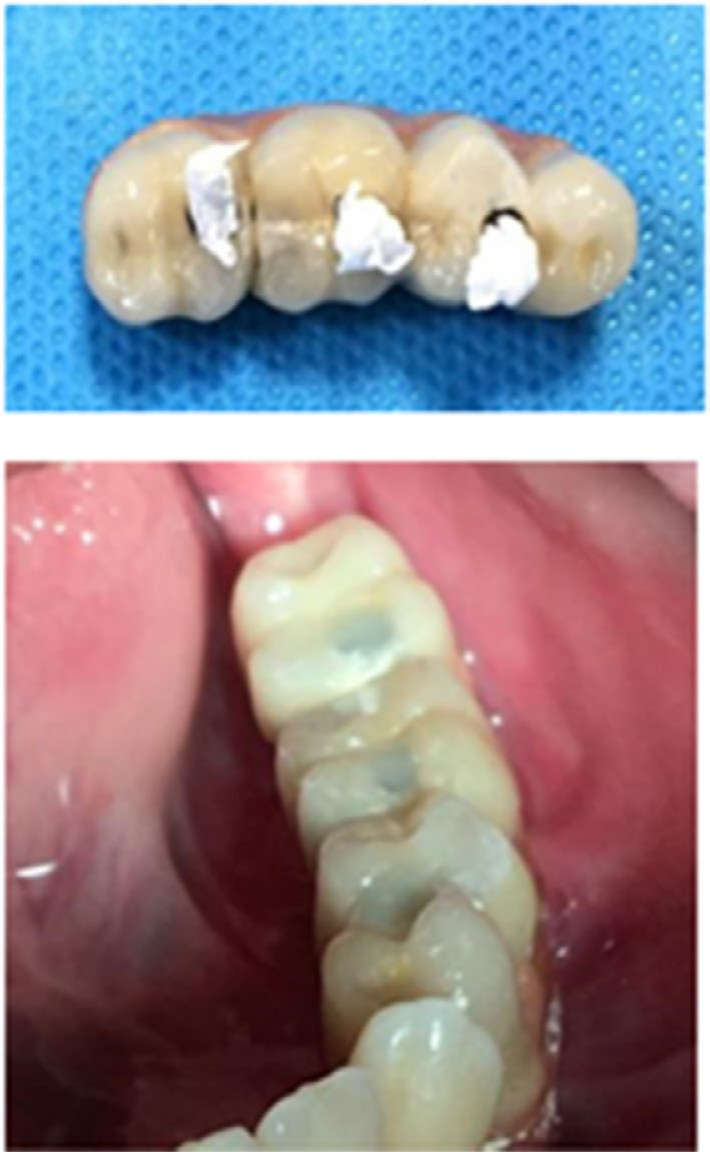
Delivery session prosthesis

**FIGURE 9:**
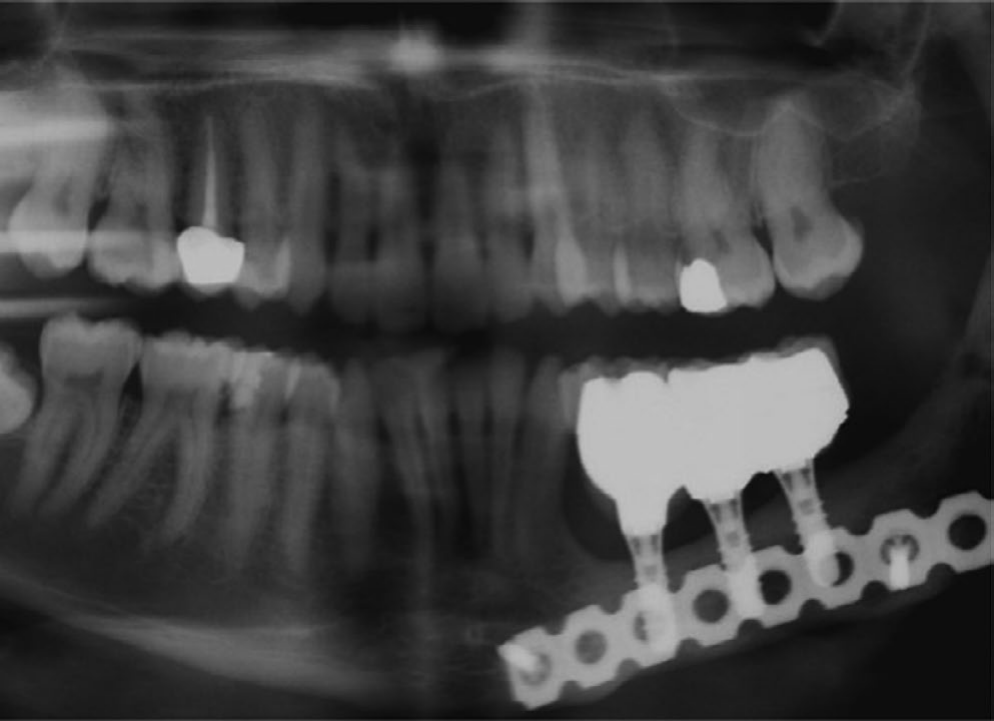
Radiograph after 3 years follow‐up

Therefore, the impression from prosthesis was prepared for providing a chairside temporary restoration. After that, the anatomic crowns were cut and picked up. It should be noted that during the anatomic crowns remaking, a provisional restoration was placed. The teflon tape was placed at the orifice of screw holes in the delivery session and after that crowns with occlusal holes were cemented with temporary cement (Temp‐Bond, Kerr). It is important to consider these access holes in our crowns because clinicians can manage some complications such as those mentioned earlier. Finally, light polymerized composite was cured for filling the holes (Figure 8). After 3 years of follow‐up, no functional or hygienic or radiographic problems were observed (Figure 9).

## 3 DISCUSSION

In the face of excessive inter‐occlusal space, there are some options such as overdenture, removable denture, and hybrid implant‐supported fixed Prosthesis. It was shown that the implant‐supported fixed prostheses provide more comfort and efficient in the mandible reconstruction, Also, considering that the removable prosthesis could increase the risk of bone graft resorption, the hybrid Prosthesis was chosen in the present cases.^11^, ^13^

The main advantage of the “Hybrid Implant Prosthesis” is that clinicians can improve implant emergence and retrievability of the bridge.^8^ Moreover, this Prosthesis reduces distortion and lack of passive fit of the framework during the porcelain firing.^14^ However, the corresponding laboratory costs are higher than other options such as an acrylic hybrid overdenture or multiple implant‐supported bridges, and some of the technical procedures like adjusting contact points are time consuming, more complex, and more expensive compared to the conventional rehabilitations.^11^

In addition, some criteria such as type of the bone, position of implants, and design of superstructures affect the success rate of implants.^15^ In order to assess the survival rate of implants, prevent continuous pain and peri‐implantitis, the mobility and radiolucency should be taken into account.^4^ All these factors were evaluated in the present cases, and none of them were observed.

The complications associated with the implant failure after the placement of prosthesis restorations have been more recorded in the case of overdentures, followed by complete‐arch fixed partial dentures and then fixed partial dentures.^16^ Some complications are common in patients such as screw loosening, decementation, and porcelain fracture.^17^ In first case, the implant which was inserted into #18 site failed prior to performing the prosthetic procedures, and due to this limitation, the cantilever forces were exerted. Another factor that contributes to the bone resorption is related to the normal growth of this patient and force factor were intensified. In the last case, screw loosening was observed, which is mainly due to factors such as method of fabrication. The present findings are consistent with results reported by Kim et al. They mentioned that the CAD/CAM custom abutment shows more misfit between abutment and screw in some cases, which may be an important factor in the screw loosening in comparison with conventional method. Hence, the manufacturer's recommendation should be noticed in CAD/CAM situations.^18^ So, given the lower complications in the conventional casting method, it is supposed to be more predictable approach.

In other case reports, conventional or CAD‐CAM methods were observed during the time. Yoon^13^ mentioned that there is no problem with the oral reconstruction by CAD‐CAM Hybrid Prosthesis during the 6‐month follow‐up. In another study, the conventional technique was applied for the fabrication of implant‐supported fixed prosthesis in patient with mandibular defect and no complication was recorded after a 1‐year follow‐up period.^19^

One of the challenges we have encountered in the first case was related to the patient's age and changes in implant positions during the jaw growth (such as infra occlusion restorations in maxilla and lingual position of implants in mandible after cease of growth). Based on a systematic review, after the permanent canine eruption in mandible, we are not concerned with the transverse growth and during growth phase there are only rotational changes that vary the position of implants compared to the adjacent teeth.^20^ One of the advantages of the Hybrid Prosthesis is that one can remove the current crowns after growth phase (if it is needed) and add porcelain in some parts while metal framework does not alter. In current case, no changes were observed in the implant positions and any possible adjustment will be evaluated in future follow‐up sessions.

## 4 CONCLUSION

The excessive space between the residual ridge and the antagonistic arch in patient who underwent mandibulectomy is considered as one of the most common problems. Based on the present achievements and follow‐up observation, the fabrication of hybrid implant restorations in these cases is reliable and durable. Also, further investigations will be needed to make comparisons between conventional and digital fabrication methods.

## CONFLICTS OF INTEREST

The authors declare that there is no conflict of interest regarding the publication of this article.

## AUTHOR CONTRIBUTIONS

Somayeh Niakan involved in prosthodontic treatment for patient, study conception and design, revision, and follow‐up. Negin Yaghoobi involved in study conception and design, wrote the manuscript, revision, and follow‐up.

## ETHICAL APPROVAL

Appropriate informed consent was taken for publication of this report and the associated images and collected in accordance with the journal's patient consent policy.

## CONSENT

Published with written consent of the patient.

## Data Availability

The data that support the findings of this study are available on request from the corresponding author. The data are not publicly available due to privacy or ethical restrictions.
